# Facile Synthesis of Defective TiO_2−x_ Nanocrystals with High Surface Area and Tailoring Bandgap for Visible-light Photocatalysis

**DOI:** 10.1038/srep15804

**Published:** 2015-10-30

**Authors:** Muhammad Wajid Shah, Yunqing Zhu, Xiaoyun Fan, Jie Zhao, Yingxuan Li, Sumreen Asim, Chuanyi Wang

**Affiliations:** 1 Laboratory of Environmental Sciences and Technology, Xinjiang Technical Institute of Physics & Chemistry; Key Laboratory of Functional Materials and Devices for Special Environments, Chinese Academy of Sciences, Urumqi 830011, China; 2 University of Chinese Academy of Sciences, Beijing, 100049, China

## Abstract

A facile hydrothermal approach has been developed to prepare defective TiO_2−x_ nanocrystals using Ti(III)-salt as a precursor and *L*-ascorbic acid as reductant and structure direction agent. The prepared TiO_2−x_ nanocrystals are composed of a highly crystallized TiO_2_ core and a disordered TiO_2−x_ outer layer, possessing high surface area, controlled oxygen vacancy concentration and tunable bandgap via simply adjusting the amount of added *L*-ascorbic acid. The defective TiO_2−x_ shows high photocatalytic efficiency in methylene blue and phenol degradation as well as in hydrogen evolution under visible light, underlining the significance of the present strategy for structural and bandgap manipulation in TiO_2_-based photocatalysis.

TiO_2_ is one of extensively studied photocatalytic materials due to its excellent physicochemical properties as well as its earth abundance, nontoxicity and stability^1^,^2^,^3^,^4^. However, the large band gap (3.2 eV for anatase) limits its utilization of sunlight and thus its practical applications in many important fields such as photocatalytic hydrogen evolution^5^, environmental remediation^6^ and solar energy conversion^7^. To overcome this barrier, great efforts have been devoted to engineer TiO_2_′s band gap from a variety of aspects in order to approach a high photoactivity under visible light irradiation. Introducing dopants is one of the most intensively investigated strategies to enhance its visible light utilization. Initially, metal ions were used as dopants to introduce states into the TiO_2_ band gap^8^,^9^,^10^, but the subsequential problems such as thermal instability, increased carrier recombination centers^11^,^12^, and the need for an expensive ion-implantation facility significantly limit its applications. Nonmetal elements was also adopted^13^,^14^, if doped under the right conditions, can effectively narrow band gap of TiO_2_ and improve its visible-light absorption. Compared to other elements, nitrogen doping is thought to be the most useful choice to enhance its visible-light photocatalytic activity^15^. But unfortunately, further studies find that the activity of N-TiO_2_ for visible-light induced hydrogen evolution is quite low^16^.

Recently, the intrinsic defects in TiO_2_ matrix such as oxygen vacancy (Vo) and Ti^3+^ have been proved to trigger the visible-light activity of TiO_2_^17^,^18^,^19^,^20^,^21^,^22^,^23^,^24^. Chen *et al*.^17^ have reported that hydrogen thermal treatment of TiO_2_ nanoparticles can generate an amorphous layer near the surface to form defective black TiO_2−x_ nanoparticles. Such defective black TiO_2−x_ nanoparticles display excellent photoactivity and stability in photocatalytic hydrogen generation. Theoretical calculations demonstrate that high vacancy concentration could induce a vacancy band of electronic states just below the conduction band and narrow the band gap to 1.0 eV. Except its optical and electronic properties, the performance of defective TiO_2−x_ also depends largely on its morphological and structural properties^25^,^26^,^27^, like its surface area^28^, particle size and pore structure^29^. However, to the best of our knowledge, rare work has been reported on controlling optical and morphological structure properties simultaneously for defective TiO_2−x_. Herein, we report a facile hydrothermal approach to produce defective TiO_2−x_ nanocrystals with high surface area and tailoring band gap using TiCl_3_ as a precursor and *L*-ascorbic acid as reductant and structure direction agent.

## Results

**Figure 1:**
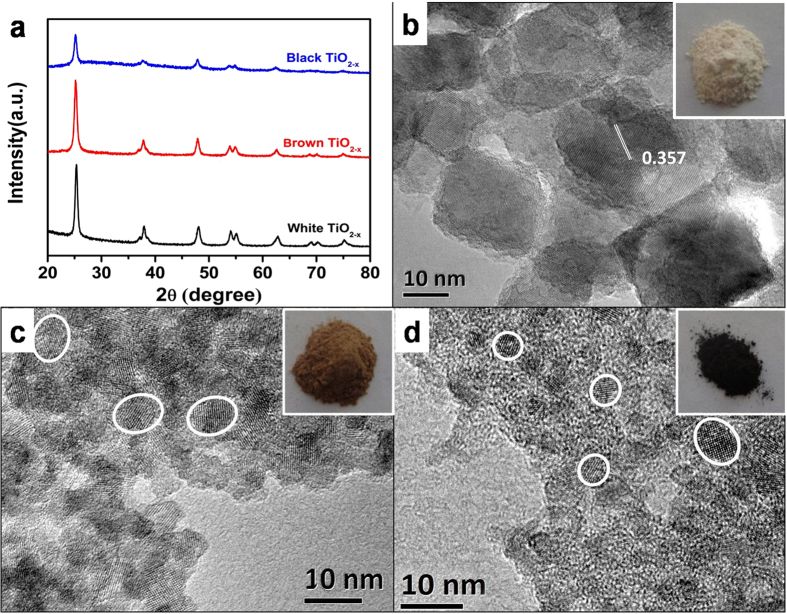
XRD patterns (a), TEM images (white TiO_2−x_(b), brown TiO_2−x_(c), black TiO_2−x_(d)), of the defective TiO_2−x_ nanocrystals.

The obtained defective TiO_2−x_ samples display diffraction peaks at 25.4°, 37.9°, 48.1° and 53.1° in the XRD patterns, indicating that as synthesized TiO_2−x_ is in a pure anatase phase (Fig. 1a). It suggests that the presence of *L*-ascorbic acid does not influence the crystal structure of TiO_2−x_. TEM analysis with the obtained defective TiO_2−x_ samples shows that the particle size of highly crystallized TiO_2_ core gradually decreases from 50 nm to 10 nm (Figs. 1b-1d) as increasing the amount of *L*-ascorbic acid from 0, 0.3 to 0.7 g in 80 mL solution. The crystal lattices of TiO_2_ core can be clearly identified in all the samples, indicating that the TiO_2_ core is highly crystallized. Since the particles are overlapped, the disordered TiO_2−x_ outer layer cannot be distinguished in TEM, but its presence can be indirectly confirmed via further sintering of the defective black TiO_2−x_ at 500 °C in N_2_ atmosphere considering that high concentration of vacancy could significantly reduce the melting temperature of TiO_2_^25^,^26^. The sintered TiO_2−x_ exhibits a nanoscroll structure with several layers of TiO_2−x_ sheets and the interlayer spacing is 0.76 nm (see Fig. S1a for details). EDX measurement (Fig. S1b) confirms that the TiO_2−x_ nanoscroll is only composed of Ti (56.4 at%) and O (28.8 at%) elements, implying the presence of defective TiO_2−x_ layer surrounding the TiO_2_ core. It is worth to mention, the defective TiO_2−x_ nanosheet is a fascinating structure to apply in Li-ion battery and photocatalysis for its ultrahigh cycle rate and high efficiency^30^,^31^. This report provides a new and simple method for preparing the defective TiO_2−x_ nanosheets.

**Figure 2:**
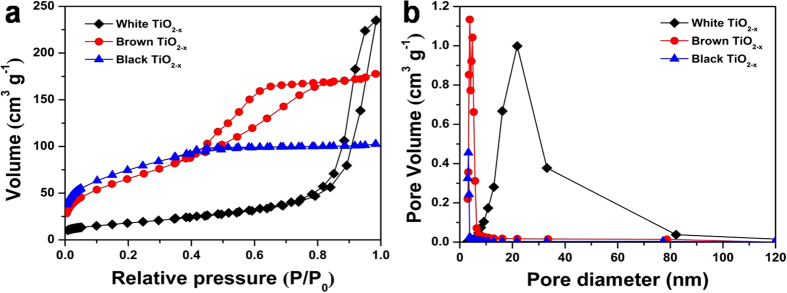
N_2_ adsorption–desorption isotherms (a) and pore size distribution (b) of the defective TiO_2−x_ nanocrystals.

The N_2_ adsorption–desorption isotherms (Fig. 2a) of defective TiO_2−x_ samples exhibit a typical type-IV isotherm with a distinct hysteretic loop, indicating mesoporous features. The average pore size (Fig. 2b) decreases with increasing the amount of added *L*-ascorbic acid, while the corresponding BET surface area (Tab. S1) dramatically increases from 64.56 m^2^ g^−1^ (white TiO_2−x_), 188.75 m^2^ g^−1^ (brown TiO_2−x_) to 263.95 m^2^ g^−1^ (black TiO_2−x_). Therefore, the presence of *L*-ascorbic acid molecules significantly affects the pore structure and surface area of the obtained TiO_2−x_ samples. The porous structure makes the defective TiO_2−x_ materials suitable for photocatalysis application because of their abundant porous channels.

**Figure 3:**
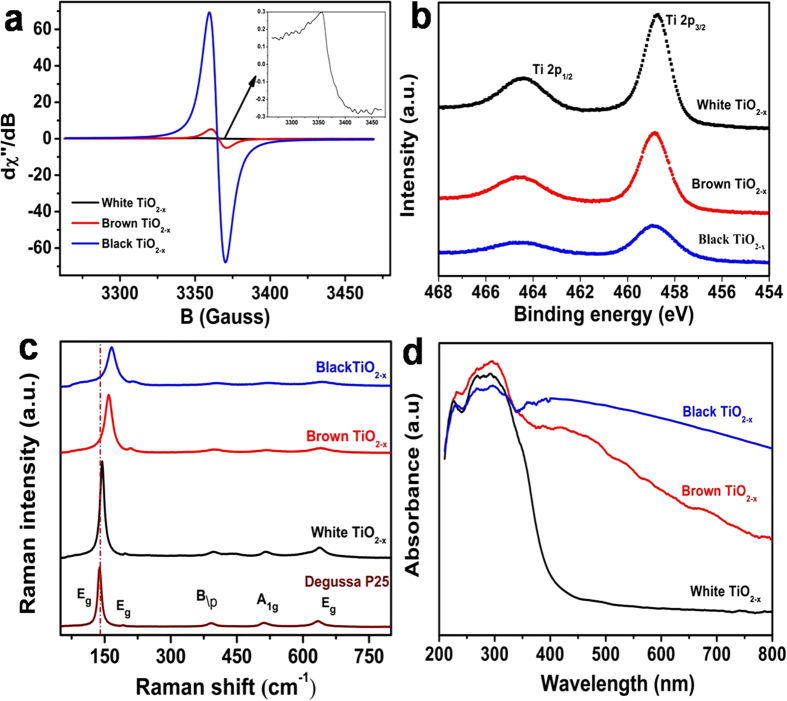
EPR (a), XPS (b), Raman (c), and DRS (d) profiles of the defective TiO_2−x_ nanocrystals.

Electron paramagnetic resonance (EPR) measurements were conducted at room temperature to verify the presence of high concentration Vo. As shown in Fig. 3a, all the defective TiO_2−x_ samples show a very strong EPR signal at *g*-value of 2.003 which indicates the significant presence of Vo. As discussed previously, the EPR signal appearing at *g*-value of 2.003 is caused by electrons trapped on surface Vo^32^,^33^. However, the representative signal of Ti^3+^ usually appearing at g ≈ 1.94^34^,^35^ is not shown here, which suggests the absence of rhombic Ti^3+^ in defective TiO_2−x_ samples. Furthermore, the EPR signals of brown and black TiO_2−x_ show a great enhancement in the intensity, which indicates their substantially increased Vo concentrations due to the increase in the amount of added *L*-ascorbic acid. Therefore, the defects in the colorful TiO_2−x_ are present as Vo instead of Ti^3+^. X-ray photoelectron spectroscopy (XPS) characterization of the white, brown and black TiO_2−x_ samples (Fig. 3b) shows only Ti2p_1/2_ and Ti2p_3/2_ peaks with slightly difference but no Ti^3+^ peaks appeared, which further confirms that Ti^3+^ does not exist in the defective TiO_2−x_ nanocrystals. Structural properties of the obtained TiO_2−x_ samples were further examined by measuring Raman scattering. For comparison, P25 Degussa was also analyzed. As shown in Fig. 3c, the six (3Eg + 2B1g + A1g) Raman-active modes of anatase phase with frequencies at 144, 197, 399, 515, 519 (superimposed with the 515 cm^–1^ band), and 639 cm^–1^ were detected in all investigated samples. Compared with P25, the defective TiO_2−x_ nanocrystals display a varying degree of blue-shift in Raman bands (Eg from 139 to 144, 153 and 164 cm^−1^, respectively), which indicates that the original symmetry of TiO_2_ lattice is broken down due to the disordered TiO_2−x_ layer formed by introducing of *L*-ascorbic acid^36^. In addition, the peak shift of defective TiO_2−x_ gradually increases along with the darkening color, suggesting the increase in the Vo concentration. This result further supports that the Vo concentration could be controlled by the amount of *L*-ascorbic acid directly. The optical property of all the defective TiO_2−x_powder samples was characterized by UV-visible diffuse reflectance spectra (DRS), as shown in Fig. 3d. The white TiO_2−x_exhibits a strong absorption in the UV range, while the brown and black TiO_2−x_ samples display a broad absorption over the entire UV-vis wavelength range investigated. Furthermore, the black TiO_2−x_ sample shows even higher absorption of UV-vis light than the brown TiO_2−x_ sample which further confirms the assumption that the high concentration of Vo is capable of generating a new vacancy band locating just below the conduction band edge of pure TiO_2_.

**Figure 4:**
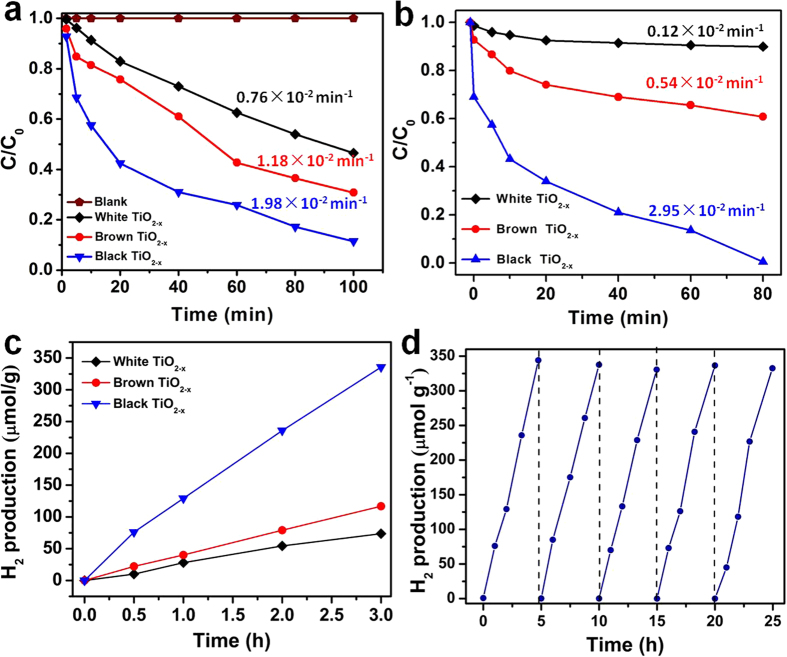
Degradation of MB (a) and phenol (b), hydrogen evolution (c) and cycle test (d) of the defective TiO_2−x_ nanocrystals under visible light (>420 nm).

**Figure 5:**
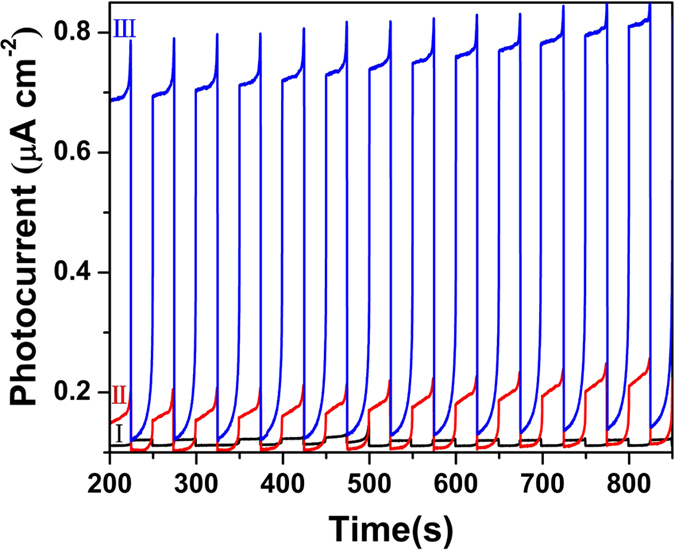
Photocurrent (I: white TiO_2−x_; II: brown TiO_2−x_; III: black TiO_2−x_) of the defective TiO_2−x_ nanocrystals under visible light (>420 nm).

**Figure 6:**
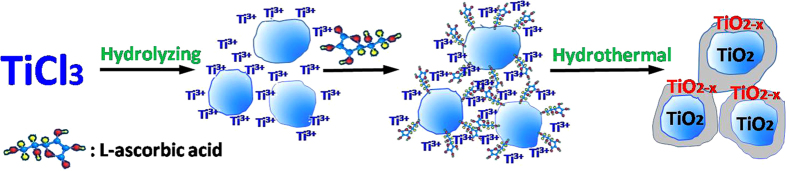
Schematic diagram for the formation of defective TiO_2−x_ nanocrystals.

The photocatalytic degradation of MB (20 mg/L) and phenol (10 mg/L) was performed using 0.5 g/L of the as-synthesized white, brown and black TiO_2−x_ powders under a 300 W Xenon lamp with UV cut-off filter (λ > 420 nm). As shown in Figs. 4a-4b, the black TiO_2−x_ exhibits the highest efficiency in both MB and phenol degradation. The kinetic reaction rate of MB degradation at black TiO_2−x_ is 1.98 × 10^−2^ min^−1^, while it is 1.18 × 10^−2^ min^−1^ at the brown TiO_2−x_, and 0.76 × 10^−2^ min^−1^ at the white TiO_2−x_. Similar improvement was also observed for phenol degradation, the kinetic rate at black TiO_2−x_is 2.95 × 10^−2^ min^−1^, which is about 25.41 times greater than that at white TiO_2−x_. The phenol molecules could be totally decomposed at black TiO_2−x_ nanocrystals under visible light irradiation in about 80 min. Moreover, the photocatalytic activity of the defective TiO_2−x_ was also tested for hydrogen generation under visible light (Fig. 4c). All the catalysts were loaded with 0.6 wt% Pt, and methanol was used as sacrificial agent. The black TiO_2−x_ nanocrystals show a much higher photocatalytic activity with a hydrogen evolution rate of 116.7 μmol g^−1^ h^−1^ compared to the white TiO_2_ (20.6 μmol g^−1^ h^−1^) and brown TiO_2−x_ (38.9 μmol g^−1^ h^−1^) under visible light irradiation in the presence of 20 mg/L photocatalyst. The cycling test results of the visible-light driven photocatalytic activity of black TiO_2−x_ nanocrystals for hydrogen evolution as a function of time during a 25-hour testing period are shown in Fig. 4d. No noticeable decrease in H_2_ production rate for black TiO_2−x_ in 5 cycle tests were observed within the test period, indicating good stability of the black TiO_2−x_ nanocrystals in the photocatalytic production of hydrogen from water under visible light. The photocatalysis results confirm that the presence of defective TiO_2−x_ outer layer with well-developed porosity is the key factor leading to improved photoactivity. The high Vo concentration in the defective TiO_2−x_ nanocrystals enhances the absorption of visible light and thereby the generation of charge carriers, which are further transformed into abundant active species of ·O_2_^−^ and ·OH to degrade the pollutants and split water to produce H_2_ under the visible light irradiation. The high surface area and rich pore structure increase the collision possibility of the pollutant molecules with catalyst surface and the adsorbed active radicals. These factors are responsible for the enhancement of photocatalytic activity of defective TiO_2−x_ nanocrystals for pollutants degradation and hydrogen evolution. This is further confirmed by the measurement of photocurrent densities with the defective TiO_2−x_ nanocrystals photoanodes at a constant potential of 0 V (vs Ag/AgCl) under visible light (Figs. 5 and 6). The photocurrent density of black TiO_2−x_ is the highest in all the samples, which is almost 10 times of that of white TiO_2−x_. Thus, the high concentration of Vo defects gives rise to high visible-light induced photo-electron transformation and results in high efficiency in photocatalytic activity.

## Discussion

Due to its unique optical property and superior visible-light-driven photocatalytic activity, defective TiO_2−x_, has attracted plenty of attentions recently. But most of the reports focused on tailoring the photochemical properties, rare study is reported on its structure and morphology. To the best of our knowledge, the highest surface area of defective TiO_2−x_ reported was near 90 m^2^/g^37^. In this report, we presented a novel method for fabricating defective TiO_2−x_ nanocrystals with tunable bandgap and high surface area using Ti(III)-salt as a precursor and *L*-ascorbic acid as reductant and structure direction agent. The formation of defective TiO_2−x_ nanocrystals is schematically shown in Fig. 5. During hydrolysis, the -TiOH^2+^ was formed first (Eq. 1) and oxidized by the dissolved oxygen to the Ti(IV)-oxo species (Eq. 2)^38^, and *L*-ascorbic acid molecules were adsorbed on the initial particle surface through Ti-O-C bond, while the excessive Ti^3+^ was diffused in the interspace of *L*-ascorbic acid molecules. The Ti(IV)-oxo species is assumed to be an intermediate between TiO^2+^ and TiO_2_, consisting of partially dehydrated polymeric Ti(IV)hydroxide^39^. Following hydrothermal process, Ti(IV)-oxo was transferred to highly crystallized TiO_2_ (Eq. 3). If without the presence of *L*-ascorbic acid molecules, the excessive Ti^3+^ would react with the dissolved oxygen molecules in the solution to grow into TiO_2_ crystals. But the chemical adsorption of *L*-ascorbic acid inhibited the diffusion of dissolved oxygen molecules to the TiO_2_ core surface, and hindered the growth of TiO_2_ crystals^40^,^41^. Since the oxidation process suffered from the insufficient supply of oxygen, after removing the adsorbed *L*-ascorbic acid, Vo was produced outside of the TiO_2_ core^20^. Therefore, a defective, nonstoichiometric TiO_2−x_ layer was formed surround the TiO_2_ core with rich oxygen vacancies. In addition, abundant pore structures were formed in the defective TiO_2−x_ layer after removing the adsorbed *L*-ascorbic acid, evidencing its critic role as a structure direction agent.


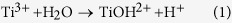



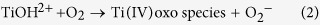



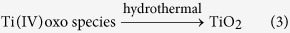


In summary, a facile hydrothermal approach has been developed for preparing defective TiO_2−x_ nanocrystals with TiCl_3_ as a precursor. *L*-ascorbic acid plays critical role in controlling the morphology and bandgap structure towards engineering the prepared defective TiO_2_. The defects in the defective TiO_2−x_ nanocrystals proved to be of Vo, while the Vo concentration and band gap of the defective TiO_2−x_ nanocrystals could be easily tailored by varying the introduced amount of *L*-ascorbic acid. Comparing with the white and brown defective TiO_2−x_, the black TiO_2−x_ shows much higher surface area and efficiency in degradation of organic pollutants (MB and phenol) and hydrogen evolution under visible light irradiation. The present work provides an alternative approach for fabricating defective TiO_2−x_ nanocrystal photocatalysts with controllable band gap and morphological structure for environmental remediation and solar fuel generation.

## Method

### Materials synthesis

For the preparation of reduced TiO_2_ nanocrystals, different amounts of L-ascorbic acid (0, 0.3 g and 0.7 g) were added to 70 mL DI water and stirred for 10 min at RT. Subsequently, 3.1 mL of TiCl_3_ was added and a purple solution was formed. Then, NaOH solution (1 mol/L) was added to raise the pH to 4. After stirring for another 30 min at RT, the mixture was transferred to a 100 mL Teflon-lined stainless steel autoclave and heated at 180 °C for 12 h. The obtained precipitates were collected by centrifugation, rinsed with water and ethanol for several times. After drying at 80 °C for overnight, the defective TiO_2−x_ samples were labeled according to its color as white, brown and black TiO_2−x_.

### Characterization

X-ray diffraction (XRD) patterns of the samples were collected on Bruker D8 Advance powder diffractometer over scattering angles from 20° to 80° using Cu Kα radiation. Transmission electron microscopy (TEM) characterization was performed on a JEOL-JEM 2100 electron microscope. Optical property was examined by UV−Visible diffuse reflectance spectrophotometer (DRS) (Shimadzu SolidSpec-3700DUV). The electron paramagnetic resonance (EPR) spectra were characterized with Bruker E500 Spectrophotometer. X-ray photoelectron spectra (XPS) of the samples were measured using a Kratos Analytical AMICUS XPS instrument.

### Methylene blue (MB) and phenol degradation

40 mL MB (20 mg/L) solution or phenol solution (10 mg/L) was placed in a 50 mL quartz photoreactor. The photocatalyst (0.5 g/L) was dispersed into the solution at neutral pH. In order to attain adsorption-desorption equilibrium, the solution was stirred in dark for 40 min. The solution was then irradiated by a 300 W Xenon lamp with UV cut-off filter (λ > 420 nm) at RT. Samples were taken at given time interval to test the concentration of MB and phenol. The concentration of MB was measured by UV‒visible spectrophotometer (UV-1800, Shimadzu). The phenol concentration was determined by Thermo Fisher Ultra 3000 HPLC equipped with a 25 cm × 4.6 mm Cosmosil C18 column.

### Photocatalytic hydrogen evolution

The photocatalytic reactions of H_2_ evolution were carried out in a closed gas circulation system with an external-irradiation type of a glass reactor. The light source was a 300 W Xenon lamp with UV cut-off filter (λ > 420 nm). The co-catalyst Pt was loaded by an in-situ photodeposition method. The 0.6 wt% of Pt-loaded catalyst (25 mg) was dispersed with a magnetic stirrer in a methanol aqueous solution (10 mL of CH_3_OH and 90 mL of H_2_O). The evolved gas including H_2_ was analyzed using an online gas chromatograph (7890A, Agilent) equipped with a thermal conductivity detector (TCD).

### Photocurrent measurement

The photocurrent was performed with an electrochemical instrument CHI660E using a three-electrode system. The samples (0.1 g) were loaded on conductive surface of ITO glass and 0.5 M Na_2_SO_4_ solution was used as electrolyte. 300 W Xenon lamp equipped with UV cut‒off filter (λ > 420 nm) was used as light source, and standard calomel electrode (SCE) as reference electrode, Pt slice as counter electrode.

## Additional Information

**How to cite this article**: Wajid Shah, M. *et al*. Facile Synthesis of Defective TiO_2-x_ Nanocrystals with High Surface Area and Tailoring Bandgap for Visible-light Photocatalysis. *Sci. Rep*. **5**, 15804; doi: 10.1038/srep15804 (2015).

## Supplementary Material

Supplementary Information
