# Supplementary material for: Facile Synthesis of Defective TiO2−x Nanocrystals with High Surface Area and Tailoring Bandgap for Visible-light Photocatalysis
Source: Sci Rep. 2015 Oct 30;5:15804. doi: 10.1038/srep15804 (PMC4626796; doi:10.1038/srep15804)
Supplement: Supplementary Information [file srep15804-s1.doc]

**Supplementary information**

**Facile Synthesis of Defective TiO_2-x_ Nanocrystals with High Surface Area and Tailoring Bandgap for Visible-light Photocatalysis**

Muhammad Wajid Shah,^a,b^ Yunqing Zhu,*^a^ Xiaoyun Fan,^a^ Jie Zhao,^a^ Yingxuan Li, ^a^ Summreen Asim^a,b^ and Chuanyi Wang*^a^

^a^ Laboratory of Environmental Sciences and Technology, Xinjiang Technical Institute of Physics & Chemistry; Key Laboratory of Functional Materials and Devices for Special Environments, Chinese Academy of Sciences, Urumqi 830011, China.

^b^ University of Chinese Academy of Sciences, Beijing, 100049, China.

**Supplemental figures.**

**
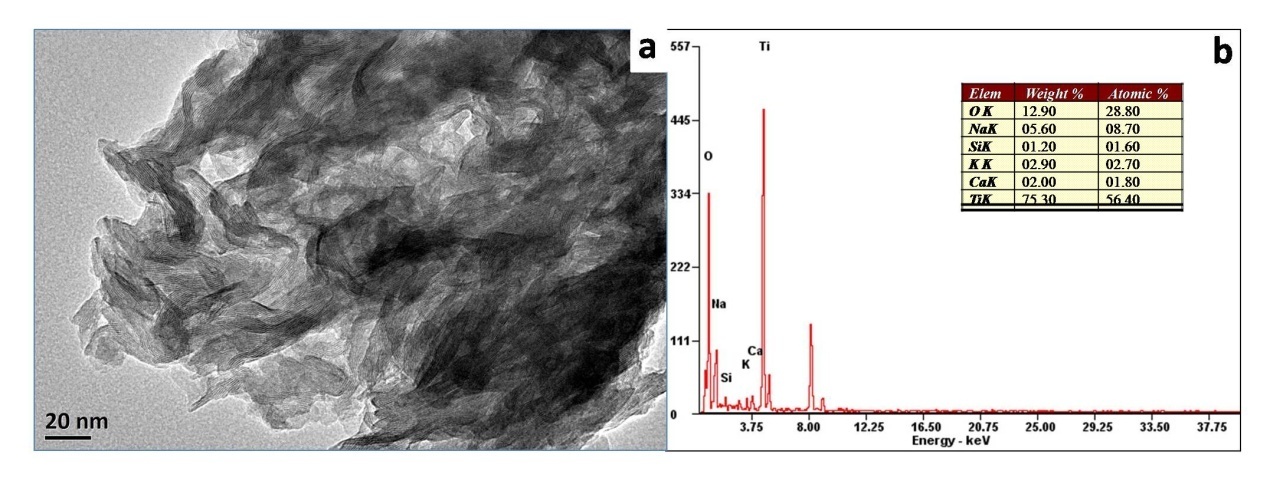
**

**Figure S1 SEM image and EDX pattern of TiO_2-x_ nanosheets.**

Table S1 Physiochemical properties of the defective TiO_2-x_ nanocrystals

| Catalysts | BET surface area  (m^2^/g) | Pore volume  (cm^3^/g) | Pore diameter  (nm) |
| --- | --- | --- | --- |
| White TiO_2-x_ | 64.56 | 0.36 | 22.58 |
| Brown TiO_2-x_ | 188.75 | 0.28 | 5.84 |
| Black TiO_2-x_ | 263.95 | 0.16 | 2.41 |
